# The Fas/CD95 Receptor Regulates the Death of Autoreactive B Cells and the Selection of Antigen-Specific B Cells

**DOI:** 10.3389/fimmu.2012.00207

**Published:** 2012-07-25

**Authors:** Gabor Koncz, Anne-Odile Hueber

**Affiliations:** ^1^Immunology Research Group of the Hungarian Academy of Sciences, University Eötvös LorandBudapest, Hungary; ^2^Institut de Biologie de Valrose, UMR CNRS 7277, UMR INSERM 1091, Université de Nice-Sophia-AntipolisNice, France

**Keywords:** cell death, immunity, survival signal

## Abstract

Cell death receptors have crucial roles in the regulation of immune responses. Here we review recent *in vivo* data confirming that the Fas death receptor (TNFSR6) on B cells is important for the regulation of autoimmunity since the impairment of only Fas function on B cells results in uncontrolled autoantibody production and autoimmunity. Fas plays a role in the elimination of the non-specific and autoreactive B cells in germinal center, while during the selection of antigen-specific B cells different escape signals ensure the resistance to Fas-mediated apoptosis. Antigen-specific survival such as BCR or MHCII signal or coreceptors (CD19) cooperating with BCR inhibits the formation of death inducing signaling complex. Antigen-specific survival can be reinforced by antigen-independent signals of IL-4 or CD40 overproducing the anti-apoptotic members of the Bcl-2 family proteins.

## Introduction

Apoptosis may be mediated by intrinsic or extrinsic mechanisms. Different stimuli such as irradiation, drugs, cytokine deprivation, DNA damage, anoikis, ER stress, etc., lead to the permeabilization of the mitochondrial membrane, activating the intrinsic pathway. This intrinsic pathway is mainly regulated by the balance and interaction of the pro- and anti-apoptotic proteins of the Bcl-2 family. The combined effect of these different factors determines the amount and the ratio of various members of the Bcl-2 family and regulates mitochondrial depolarization (Elmore, 2007).

The extrinsic pathways are mediated by cell death receptors, including the Fas (CD95/Apo-1/TNFSR6) receptor-ligand system. Upon the engagement of the cell death receptor by FasL, Fas rapidly recruits the adapter molecule FADD and caspase-8 proenzyme via interactions with the homologous death domain (DD) and death effector domain (DED), forming the death-inducing signaling complex (DISC; Kischkel et al., 1995). This leads to processing and activation of caspase-8, the initiator caspase. Depending on the amount of activated caspase-8, this protease can activate the executioner caspases directly (Type-I cells), or indirectly through the cleavage of Bid (Type-II cells), where the truncated form of Bid (tBid) dominates the pro-apoptotic part of the Bcl-2 family, pushing the balance of anti- and pro-apoptotic proteins toward mitochondrial depolarization. In Type-II cells, Fas-induced cell death can be inhibited by the upregulation of anti-apoptotic Bcl-2 proteins, compensating the elevated tBid level. In Type-I cells, induced cell death can be blocked by the downregulation of the number of available receptor molecules (death receptors/decoy receptor ratio), by the regulation of death receptor aggregation, or by FLIP. FLIP, a caspase-8 homolog (lacking caspase-8’s protease activity), can block the FADD-caspase-8 association, inhibiting cell death receptor signaling (Scaffidi et al., 1999).

Evidence accumulated thus far suggests that, apart from the control of death signaling through the number of receptors and oligomerization, anti-apoptotic members of the Bcl-2 family can inhibit the intrinsic pathways in both cell types and only the extrinsic pathways of Type-II cells, while FLIP is able to inhibit only extrinsic pathways, but in both cell types. Pro and pre-B cells as non-activated lymphocytes die principally by intrinsic pathways, chiefly through the upregulation of the Bim pro-apoptotic factor. Accordingly, in these stages of cell development, different survival signals, such as IL7 or BCR tonic signals trigger elevated levels of A1 or Mcl-1 anti-apoptotic proteins of the Bcl-2 family (Opferman, 2008). However, upon antigen recognition, immunoreceptor triggering, cytokines or growth factors (mainly through the regulation of AKT; Suzuki et al., 2003), the Jak-STAT (Khaled and Durum, 2003) pathway, and elevated metabolism (Khan, 2009) strongly push the balance of pro- versus anti-apoptotic members of Bcl-2 family toward survival. Therefore activated lymphocytes must be relatively resistant to the intrinsic apoptotic pathway. Non-activated B cells express a minimal amount of Fas. However, upon activation, Fas expression increases. This ensures an additional and necessary break in the regulation of activated B cells. Simultaneously, with the emergence of potential Type-I cell death, FLIP-mediated blockage of Fas-induced signaling becomes crucial to prevent the unwanted death of activated B cells. With respect to the survival mechanisms against Fas-mediated cell death studied in activated B cells, the number of receptors was not intensively regulated, while the influence of survival signals on receptor oligomerization has not been systematically investigated. Our current knowledge suggests that FLIP is a critical regulator of Fas-mediated B cell death. Elevating FLIP expression results in the quasi conversion from Type-I to Type-II cells (Verbrugge et al., 2010) followed by the upregulation of different anti-apoptotic members of the Bcl-2 family that has been suggested to boost the survival effect of FLIP. In this review, we summarize the different survival signals protecting activated B cells from Fas-induced cell death.

The IAP family proteins mediate another potential mechanism that inhibits different apoptotic routes. These proteins can directly block caspase activation. Their very short half-life keeps the amount of these proteins relatively constant, ensuring the blockage of unwanted caspase activity, but allowing caspase activity from different stimuli. The irregular upregulation of any member of this family ensures the blockage of apoptosis for only a very limited period, explaining why survivals signal rarely regulate the amount of these proteins.

## Effects of B Cell Specific Fas Depletion and Fas Expression on B Cells: *In Vivo* Studies

The interaction between Fas receptor and FasL plays an essential role in the maintenance of immunological tolerance. Lack of function mutations in the Fas receptor (e.g., in *lpr* mice) or the FasL (*gld* mice; Takahashi et al., 1994) leads to immune dysfunction in association with particular genetic backgrounds. These include lymphadenopathy, splenomegaly, hyperimmunoglobulinemia, glomerulonephritis, and increased development of B lymphomas (Watanabe-Fukunaga et al., 1992). A similar phenomenon has been observed in human Autoimmune Lymphoproliferative Syndrome (ALPS; Lenardo et al., 2010), most frequently through various mutations in Fas or in other molecules implicated in Fas-mediated signaling (Fisher et al., 1995; Rieux-Laucat et al., 1995).

Historically, the most important role of Fas was reported to be the regulation of T cell activity. However cell type specific deletion of Fas highlighted its crucial role in the cell death process of different types of cells (including B cells).

Fas was thought to play the main role in T cell regulation based on the abnormal accumulation of the CD3+ B220+ CD4− CD8− T cell population in lpr mice or mice with T cell specific depletion of Fas (Stranges et al., 2007). Nevertheless, in B cell specific Fas deficient mice, splenomegaly evolved with age without the appearance of the B220+ DN T cell population (Stranges et al., 2007; Hao et al., 2008). Similarly, the accumulation of both IgM and IgG2a producing autoreactive B cells and increased serum antibody and autoantibody concentrations appear when Fas is specifically deleted in B cells. In contrast, autoantibody production did not occur in T cell specific deletion of Fas (Hao et al., 2004; Stranges et al., 2007). The elevated levels of autoantibodies result in hyperimmunoglobulinemia, vasculitis, and glomerulonephritis (Cohen and Eisenberg, 1991; Shlomchik et al., 1994; Stranges et al., 2007; Hao et al., 2008). The deposition of immune complexes in the kidney, lymphocyte infiltration, and tissue destruction in the liver and lungs leads to lethality in mice of 6–18 months of age in the absence of Fas.

In these mice, in addition to the emergence of B cells, the B cell specific depletion of Fas leads to elevated T cell numbers, as both the exaggerated MHCII expression and the augmented B cell numbers result in the increased antigen presenting capacity of B cells (Stranges et al., 2007; Hao et al., 2008). An increased number of irregular T cell–B cell interactions are formed, concentrated mainly in the T cell rich periarteriolar lymphoid sheaths of the spleen (PALS; Jacobson et al., 1995; Stranges et al., 2007; Hao et al., 2008) in lpr mice or in mice with B cell specific Fas deletion (indicating that the number of B cells is regulated mainly in the T cell rich area in normal mice).

In transgenic *lpr/lpr* mice, lymphadenopathy was still observed when functional Fas expression was restored exclusively in B cells. This is due to the expansion of B220+ T cells. However, the serum Ig level was comparable with wild type mice, while the serum level of anti-dsDNA autoantibody was even lower than in normal mice (Komano et al., 1999). Surprisingly, not only was the autoantibody titer limited but antigen-specific responses were also absent, even after secondary immunization against both T-dependent and -independent antigens (Komano et al., 1999). The enhanced death of antigen-specific B cells can be explained by the fourfold greater lytic activity of T cells in lpr mice compared to normal mice due to the higher FasL expression (Chu et al., 1995) and the elevated number of FasL+, DN B220+ T cells in lpr mice (Komano et al., 1999). (However the different localization of this T cell population and the abnormally developed B cells make us question the role played by DN B220+ T cells in cell death of B cells in *lpr/lpr* mice; Jacobson et al., 1995). The unwanted death of antigen-specific B cells by exaggerated Fas signaling was confirmed with *in vitro* experiments, where the “hyperactivation” of Fas with FcγR bound anti-Fas antibody disabled antigen receptor-mediated survival (Foote et al., 1996b).

In summary, *in vivo* data indicate that while abnormally intensive Fas/FasL interaction can prevent antigen-specific B cell responses (Komano et al., 1999), the defect in Fas function results in uncontrolled autoantibody production, autoimmunity, and increased risk of B cell lymphomas, revealing that the Fas/FasL balance must be very accurately regulated during the humoral immune response.

## B Cell Specific Depletion of FADD, Caspase-8, and FLIP

FADD and caspase-8 are indispensible in Fas-mediated apoptosis. In addition, they contribute to an inhibition of necroptosis, a regulated RIP1- and RIP3-dependent necrotic cell death form (Vandenabeele et al., 2010; Han et al., 2012). An elevated necroptosis due to FADD and caspase-8 deficiencies is associated with embryonic lethality in mouse. Thus FADD and caspase-8 deficiencies result in a different manifestation from Fas depletion. Blocking the effect of elevated necroptosis in the double knock-out of caspase-8 and RIP3 results in viable mice, reminiscent of lpr mice. These mice contain B220+ T cells and show splenomegaly and lymphadenopathy (Oberst et al., 2011) due to abnormally high level of T cells. Not all aspects of B cell response were investigated in doubly knock-out mice. B cell development was normal in RIP and FADD doubly knock-out and the B cells responded normally to stimulation with anti-IgM.

The role of FADD in B cells could not be concluded in FADD-knock-out mice due to the organism-wide deficiency. A study using B cell specific FADD knock down demonstrated that FADD depleting had no effect on B cell development in the bone marrow, but resulted in elevated B cell number in the lymphoid organs. Surprisingly the number of B1 cells was significantly reduced (Imtiyaz et al., 2006). B cells collected from FADD^−/−^ mice were resistant to recombinant FasL-induced cell death in *in vitro* culture, indicating the indispensible role of FADD in Fas-induced signaling in B cells. However, despite the essential role of FADD in Fas-induced apoptosis in B cells, mice with B cell specific FADD depletion, even aged, did not exhibit B cell-related autoimmune diseases which are characteristic of Fas deficiency. The BCR- and CD40-induced B cell proliferation was normal in these mice, whereas TLR3 and TLR4 induced proliferation were markedly decreased (Imtiyaz et al., 2006).

B cell specific depletion of caspase-8 like the absence of FADD resulted in elevated B cell number in the spleen, and reduced amount of B1 cells (Beisner et al., 2005). The BCR, CD40, and CpG-induced proliferation were unaltered, while TLR3 and TLR4-generated proliferation was dramatically reduced. Fas-mediated apoptosis was completely abrogated, but the plasma titer produced by the caspase-8-deficient B cells against either the T cell-dependent or independent antigen was unchanged (Beisner et al., 2005).

Fas-FADD chimera was constructed containing the first 183 amino acids of Fas and the DED of FADD. Expression of this protein in lymphocytes of MRL-lpr/lpr mice completely diminishes their T cell abnormalities resulting in the disappearance of the unusual B220+ T cells representative of Fas deficiencies (Kabra et al., 1999). In contrast to T cell discrepancies B cell-related anomalies were not reconstituted by Fas-FADD chimera. Reminiscent to lpr/lpr mice serum antibody level and anti-DNA autoantibody level were higher in Fas-FADD chimera transgenic mice than in normal mice. This indicates that FADD-independent Fas-mediated pathway may operate in B cells, but not in T cells.

Results observed in B cell specific FLIP-deficient mice indicate that FLIP is dispensable for the development of B cells in the bone marrow. The numbers of B cells purified from the spleen and lymph nodes of *b-cflip*-deficient mice are significantly reduced when compared with control mice. The data showed that the percentages of B1 cells in the absence of FLIP were lower than in control mice.

While FLIP-deficient B cells can be stimulated with anti-IgM or CD40 to proliferate, this proliferative response cannot be sustained due to the rapid onset of apoptosis (Coffey and Manser, 2010).

B cells lacking FLIP were more sensitive to Fas-induced apoptosis and their participation in the formation of GC responses was inhibited (Zhang et al., 2009; Coffey and Manser, 2010).

The levels of TNP-specific Ig in FLIP*-*deficient mice were lower than in control mice immunized with either the T cell-independent or the T cell-dependent antigens. In summary FLIP plays crucial role of Fas-mediated apoptosis in B cells, as well as in the regulation of B cell number, and in the survival of antibody producing B cells.

## Function of Fas in the Germinal Center

The most unambiguous appearance of Fas is within the activated B cells of germinal centers (GCs; Rathmell and Goodnow, 1995; Liu et al., 1997; Wang and Watanabe, 1999; Mizuno et al., 2003), while the expression and function of Fas on other B cells is highly questionable (Table 1). It is well accepted that Fas expression is markedly upregulated in activated germinal center B cells both in mouse and human systems. Fas expression of lymph node or spleen B cells *in vitro* and *in vivo* is markedly increased by PKW, LPS, and ConA activated T cells and mainly by the CD40L (Mandik et al., 1995; Onel et al., 1995; Rothstein et al., 1995; Wang et al., 1996).

**Table 1:** **Expression and function of Fas and sensitivity to Fas-induced cell death in different B cell populations**.

Cell type		Human	Mouse
B1 cells	Expression	Negative, CD40 stimulation upregulates Fas expression and induces a biphasic profile (Huck et al., 1998; Kodama et al., 2001)	Negative, LPS stimulation upregulates Fas expression (Mandik et al., 1995) and induces a biphasic profile (Hirose et al., 1997; reviewed in Wang and Watanabe, 1999)
	Function	Biphasic sensitivity (Kodama et al., 2001)	Hardly susceptible (Masuda et al., 1997), Function in autoantibody production in lpr mice (Watanabe et al., 2002; Qian et al., 2006) biphasic sensitivity (Hirose et al., 1997)
Mantle zone	Expression	Negative, inducible? (Leithauser et al., 1993; Moller et al., 1993; Yoshino et al., 1994; Tsunoda et al., 2000)	No data
	Function	Participation in autoantibody production (Qian et al., 2006)	No data
Pre, pro, immature	Expression	Weak (Nishiuchi et al., 1996; Nilsson et al., 2000)	Weak, positive (Mandik et al., 1995; Onel et al., 1995)
	Function	Resistant (Nishiuchi et al., 1996; Nilsson et al., 2000) debated in Lanvin et al. (2003)	Resistant (Mandik et al., 1995; Onel et al., 1995)
Blood	Expression	5–25% positive (Miyawaki et al., 1992; Daniel and Krammer, 1994), PKW (Daniel and Krammer, 1994; Yoshino et al., 1994), IL2 + aIgM induces (Huck et al., 1998)	Partially positive, LPS induces (Wang et al., 1996)
	Function	Resistant (Yoshino et al., 1994) sensitive upon upregulation (Daniel and Krammer, 1994)	Resistant (Wang et al., 1996)
Memory	Expression	Low positivity (Liu et al., 1995; Choe et al., 1996; Lagresle et al., 1996)	Low positivity (Tsunoda et al., 2000; Takahashi et al., 2001)
	Function	Resistant (Liu et al., 1995; Choe et al., 1996; Lagresle et al., 1996; high BCL-2 expression)	Resistant (Tsunoda et al., 2000; Takahashi et al., 2001; high BCL-2 expression)
Plasma cells	Expression	Tonsil: positive (Choe et al., 1996), negative (Moller et al., 1993; Merville et al., 1996) in bone marrow: weakly positive (Nishiuchi et al., 1996)	Weak positivity upon immunization in spleen (Smith et al., 1995)
	Function	Leukemia or myeloma cells unclear results (Shima et al., 1995; Sampalo et al., 2000)	No data

*For germinal center cells, see the text*.

Besides the data on Fas expression, functional investigations confirm that Fas plays a crucial role in GC B cells. Specific deletion of Fas from GC B cells produces B cell-related discrepancies documented in lpr mice or in mice with general B cell depletion of Fas, like elevated serum antibody titer, splenomegaly, lymphoadenopathy without the appearance of abnormal DN T cell population (Hao et al., 2008). The elevated B cell number was created mainly by CD38+ memory B cells rather than naive or GC B cells.

**Figure 1:**
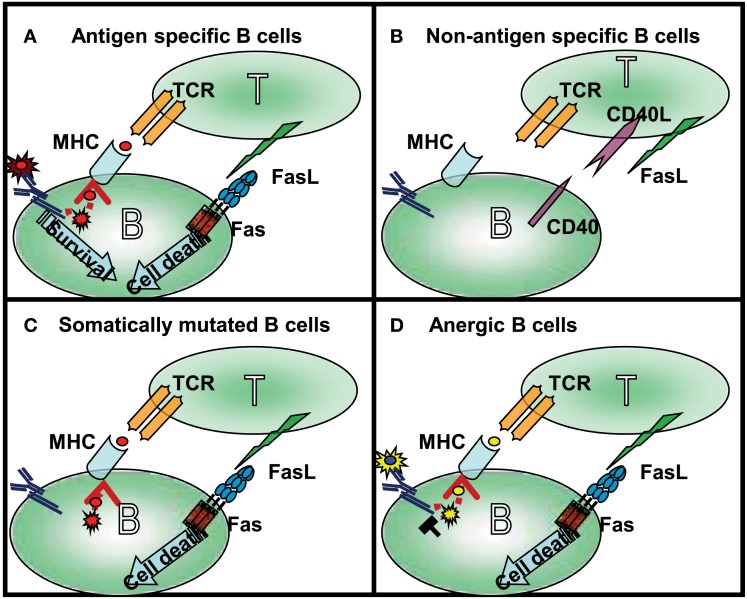
**Four different types of T cell–B cell interaction can occur in the germinal center: (A) following antigen recognition the specific B cells express Fas and in time became capable of presenting antigen to antigen-specific T cells**. Strong affinity B cells maintain their antigen specificity during somatic mutation and as a consequence the BCR transduces signals even during competition for the limited available antigen. The BCR-mediated survival signals compete with Fas-induced cell death. **(B)** The naive (bystander) B cells do not interact with activated, FasL-expressing T cells in the absence of presentation of the appropriate antigen. In this situation their Fas expression is also very limited. As mentioned above, these cells are not targets of Fas-induced cell death. **(C)** The low affinity B cells, and B cells which lost antigen-specificity because of somatic mutation, are first targets of Fas-mediated killing. Maintaining antigen presenting capacity, but losing antigen triggering in the competition for the antigen, these cells contact the T cells, but do not access the survival signal. This explains the elevated antibody level in lpr mice where the antigen non-specific B cells can survive. **(D)** Anergic B cells are the main targets of Fas-mediated killing; the antigen receptor signaling of anergic B cells is desensitized due to their permanent activation. This group can explain the enhanced *autoantibody* production observed in lpr mice.

**Figure 2:**
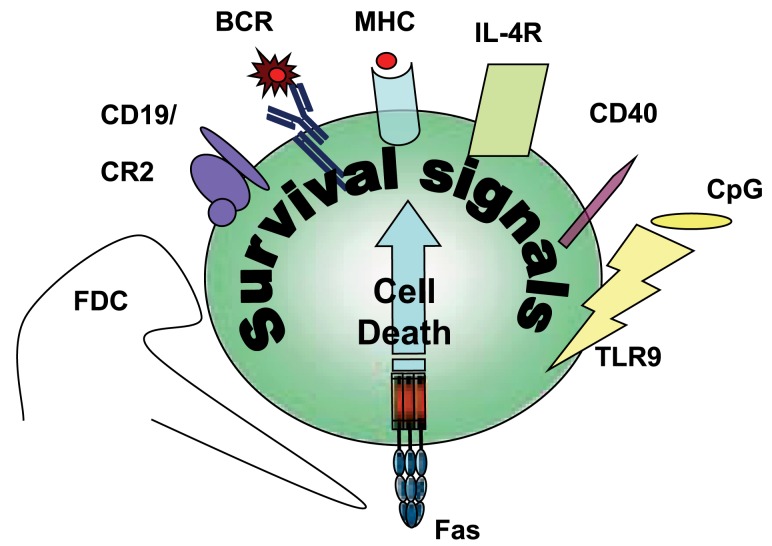
**Survival signals rescuing Fas-induced apoptosis in B cells**.

In GCs during T cell-dependent immune response, antigen recognition by B cells is linked with T cells that recognize the same antigen (Figure 1). The successful presentation of the B cell’s antigen strengthens the T cell/B cell interaction, which transduces long-term, direct signaling between the two cells. Because CD40L expression on T cells appears within a few minutes following antigen stimulation (van Kooten and Banchereau, 2000), the CD40L-CD40 receptor pair will trigger the B cells to express Fas. CD40 signals launch B cell proliferation, differentiation, germinal center development, isotype switching, etc. (reviewed in Bishop and Hostager, 2003). In addition to inducing Fas expression, CD40 signals provide a potential restraining mechanism to prevent the hyperactivity of humoral immune responses. In the following parts of this review we will summarize the survival signals rescuing the germinal center B cells from Fas-mediated cytotoxicity (Figure 2).

## Antigen-Specific Survival Signals and Inhibition of Fas-Induced Cell Death

### BCR

The FasL-induced stimulation by antigen-specific T cells can kill germinal center B cells unless a survival signal is received. The main rescue signal is transduced by BCR upon recognition of the antigen (extensively reviewed in Wang and Watanabe, 1999; Rothstein, 2000; Mizuno et al., 2003), such that Fas can only induce cell death in B cells with an impaired BCR signaling capacity or diminished antigen recognition. In accordance with the theory regarding the regulation of B cell homeostasis, the Fas receptor induces cell death in tolerant B cells upon the desensitization of BCR signaling (Goodnow, 1996) and plays a role in the elimination of the non-specific and autoreactive B cells, generated by somatic mutations that take place in the germinal center (Takahashi et al., 2001; Hao et al., 2008; Ait-Azzouzene et al., 2010) (Figure 1). This selection process will lead to the death of the non-specific or non-responsive (anergic) B cells (Rathmell et al., 1995, 1996; Rothstein et al., 1995), while specific B cells become plasma cells, memory cells, or re-enter into the germinal center cycle again.

Different signaling pathways were considered regarding the regulation of the BCR-transduced survival signals (Table 2).

**Table 2:** **Detailed molecular mechanism of inhibition of Fas-induced cell killing**.

	BCR	MHCII	CD19	CD40	IL-4	FDC	CpG
Fas expression	Unchanged/increased (**Garrone et al., 1995**; Rathmell et al., 1995; Rothstein et al., 1995; Foote et al., 1996b; Nakanishi et al., 1996; Bras et al., 1997; Schneider et al., 1997; Tumang et al., 2002; Schram and Rothstein, 2003; Moriyama and Yonehara, 2007) Decreased (Choe et al., 1996; Lagresle et al., 1996)	Not altered (Yoshino et al., 1995; Catlett et al., 2001; Blancheteau et al., 2002)	Not decreased (Barrington et al., 2005)/decreased (**Mongini et al., 2003**)	Upregulated (Garrone et al., 1995; Lagresle et al., 1995; Onel et al., 1995; Rathmell et al., 1995; Schattner et al., 1995; Wang et al., 1996)	Unchanged/increased (Foote et al., 1996a; Koizumi et al., 1996; Nakanishi et al., 1996; Wurster et al., 2002)	Upregulated (Tsunoda et al., 2000)	Decreased (Wang et al., 1997) Not decreased (Hancz et al., 2012)
DISC level	Reduced (Catlett and Bishop, 1999)/not changed (Carey et al., 2000; Wang et al., 2000) FADD association Reduced caspase-8 association (Wang et al., 2000) Reduced caspase-8 cleavage (Bras et al., 1997; Catlett and Bishop, 1999; Hinshaw et al., 2003; Moriyama and Yonehara, 2007) Transient upregulation of FLIP (Wang et al., 2000; Hennino et al., 2001; Owyang et al., 2001; Schram and Rothstein, 2003; Moriyama and Yonehara, 2007) FLIP silencing leads to Fas sensitivity (Moriyama and Yonehara, 2007) FLIP overexpression leads to resistance (Wang et al., 2000)	Reduced caspase-8 activity (Catlett et al., 2001) Elevated caspase-8 cleavage (Blancheteau et al., 2002)	Reduced caspase-8 cleavage (**Mongini et al., 2003**) Upregulation of FLIP (Mongini et al., 2003; Barrington et al., 2005)	Not changed Fadd association (Benson et al., 2006; Eeva et al., 2007) Decreased caspase-8 activation (Benson et al., 2006; Eeva et al., 2007) Flip expression is upregulated (Hennino et al., 2000, 2001; Eeva et al., 2007)		Reduced caspase-8 activity (van Eijk et al., 2001b) without FDC FLIP expression decays (Hennino et al., 2001) presence of FDC restore FLIP expression (van Eijk et al., 2001b)	Reduced caspase-8 activity (Hancz et al., 2012)
Mitochondria	Transient upregulation of Bcl-xl (Choe et al., 1996; Koizumi et al., 1996; Schneider et al., 1997; Owyang et al., 2001; Tumang et al., 2002; Schram and Rothstein, 2003) Bfl-1 upregulation (Tumang et al., 2002) Effective survival without upregulation of Bcl-xl (**Alam et al., 1997**; Owyang et al., 2001; Hinshaw et al., 2003; Moriyama and Yonehara, 2007) Overexpression of Bcl-xl does not display absolute survival (Schneider et al., 1997)		Upregulation of Bcl-2 (Mongini et al., 2003) Bcl-2 was not changed in (Barrington et al., 2005)	Bfl-1, A1, Bcl-xl upregulated (Lee et al., 1999)	Upregulated Bcl-xl (Wurster et al., 2002) marginal Bcl-xl upregulation (Schneider et al., 1997)	Bcl-2, Bcl-xl, Bax amount was unchanged (Schwarz et al., 1999; Tsunoda et al., 2000)	
Other	Upregulation of FAIM, which results in the increase of FLIP expression (Schneider et al., 1999; Owyang et al., 2001; Huo et al., 2009)			Gadd45β upregulation (Zazzeroni et al., 2003)			

*Bold font, human; normal font, mouse*.

***Human: BCR** (Garrone et al., 1995; Choe et al., 1996; Lagresle et al., 1996; Hennino et al., 2001; Alam et al., 1997), **MHC** (Yoshino et al., 1995; Blancheteau et al., 2002), **CD19** (Mongini et al., 2003), **CD40** (Garrone et al., 1995; Lagresle et al., 1995; Schattner et al., 1995; Benson et al., 2006; Hennino et al., 2000; Lee et al., 1999; Hennino et al., 2001), **FDC** (Tsunoda et al., 2000; van Eijk et al., 2001b; Hennino et al., 2001)*.

### MHC II

Beside antigen recognition by B cells, TCR-MHC interaction requires the specificity of the adaptive immune response. B cells are effective antigen presenting cells capable of initiating cell–cell interactions with T cells. In the B cell–T cell pair TCR-MHC-interactions ensure the common specificity of the two cells, guaranteeing that the correct antigen is presented to the specific T cells. Without BCR-mediated uptake, processing, and presentation of the adequate antigen by B cells long-term B cell–T cell linkage cannot be created. The prolonged T cell–B cell interaction also increases the potential Fas-FasL interaction time. The importance of the antigen presentation in Fas-mediated cell death of B lymphocytes was clearly confirmed when peptide fragments of HEL antigen were loaded into B cells. Since B cells recognized only the whole antigen and not the fragments the BCR-transduced survival was inactive, but the antigen presenting capacity remained normal with respect to the peptide specific T cells. In this model, antigen presenting B cells were killed by the HEL specific T cells. Without the peptide loading (without the antigen presenting capacity), the B cells survived (Rathmell et al., 1996; Janssens et al., 2003).

Beyond the ability to trigger the T cells, MHCII is capable of transducing its own (reverse) signals, and consequently triggering B cell activation (Scholl and Geha, 1994; Al-Daccak et al., 2004). Several studies investigated the effect of MHCII-mediated signaling on Fas-induced apoptosis, but the impact of MHCII-transduced signals on Fas-induced cell death is debated. Using different cell lines, pre-treatment with agonistic anti-MHCII antibodies before anti-Fas addition enhances the cell death intensity in case of immature and blastoid B cell lines, but has no effect on plasmacytic cell lines (Yoshino et al., 1995; Blancheteau et al., 2002). In contrast, results obtained with mouse spleen B cells and the A20 B cell line indicate that simultaneous MHCII signals with Fas triggering protect the B cells from Fas-stimulated cell death (Catlett et al., 2001), but 24–96 h pre-treatment of anti-MHCII increased the Fas-mediated cell death, at least partially because, surprisingly, it induced FasL expression on the B cells (Truman et al., 1997).

Due to the divergent functional data, conflicting results have been published concerning the effect of MHCII on the Fas-mediated signaling pathway (Table 2). In A20 mouse B cell line, the MHCII signal had no influence on Fas/Fadd association, but reduced the caspase-8 activity and cell death (Catlett et al., 2001). In contrast on LAD human B cell line, a 30-min pre-treatment of MHCII by enhancing the FADD recruitment to the Fas receptor, augments the caspase cleavage and cell death (Blancheteau et al., 2002). Regrettably all above experiments were performed by usage of anti-MHCII antibodies, without studying the direct T cell–B cell connection.

### Coreceptors

Accumulated evidence suggests that other signals that are not directly implicated in antigen recognition may modify the BCR-mediated survival. CD19, a positive coreceptor of the BCR, reduces the required threshold for B cell activation. Published data indicate that either positive (CD19) or negative coreceptors (FcγRIIb, CD22) modify the strength or the threshold of the BCR signaling. Among them, the effect of CD19, the signaling part of complement receptor 2 (CD21) was studied as a regulator of Fas-induced cell death. It is well known that coactivation of BCR with CD19 reduces the threshold of anti-IgM signaling 10- to 100-fold (Carter and Fearon, 1992), attracting attention to the importance of the complement system in the immune complex. In accordance with these results, recombinant antigen fused with the C3d_3_ complement component reduced 100-fold the amount of antigen necessary for 50% protection against anti-Fas-induced cell death (Barrington et al., 2005). Further confirming the role of complement fragments in survival signals, increased apoptosis was observed in CR2^−/−^ B cells, but not in CR2^−/−^ lpr mice (Barrington et al., 2005). The intensively triggered CD21 alone is capable of generating partial survival (Mongini et al., 2003) and co-stimulation of the anti-CD21 with anti-IgM enhancing protection against Fas-induced cell death (Mongini et al., 2003). The exact signaling of this effect is not clear (Table 2). Coactivation of CD21 and BCR in human tonsillar B cells decreased Fas expression upon CD40 stimulus, increase the levels of anti-apoptotic Flip and Bcl-2 (but not BCL-xl) and reduced caspase-8 cleavage when compared to BCR activation alone (Mongini et al., 2003) However activation of mouse B cells by recombinant antigen fused with C3d_3_ resulted in only an increase in expression level of FLIP but not Bcl-2. Similarly antigen stimulation of CR2^−/−^mice resulted in normal Fas expression and reduced amount of FLIP but not BCL-2 (Barrington et al., 2005).

## Non-Antigen-Specific Survival Signals

Antigen-independent (non-specific) B cells, in which activating signals do not markedly alter the balance of anti- and pro-apoptotic proteins toward survival, can be a target of Bcl-2 regulated escape from Fas-induced cell death.

### CD40

CD40 is one of the most important coreceptors of B cells, playing a crucial role in the stimulation of B cell proliferation, inducing somatic mutation and class switch. The main source of the B cell stimulating CD40L is derived from activated T cells, but dendritic cells and germinal center B cells (Grammer et al., 1995, 1999) also carry the ligand. Upon triggering, CD40 upregulates Fas expression in both naive, germinal center, and memory B cells and facilitates the apoptotic death of stimulated cells. CD40L is fully involved in the induction of Fas in anergic B cells. In specific B cells, due to the more intensive BCR signaling, CD40L is only partially responsible for Fas upregulation (Rathmell et al., 1996). Besides Fas upregulation, the CD40L-induced stimulus, alone (Cleary et al., 1995; Hennino et al., 2000), or synergistically with the BCR signal (Koopman et al., 1997; Lee et al., 1999; Hennino et al., 2000; Zazzeroni et al., 2003; Ho et al., 2005; Kater et al., 2005; Benson et al., 2006; Eeva et al., 2007), provides a short term (1 to maximum 2 days) rescue signal against Fas-induced death (reviewed in Guzman-Rojas et al., 2002). Several publications explain this survival signal (Table 2).

1. The assembly of DISC in mouse and human B cell lines is regulated by CD40 signals. Resembling the BCR-mediated survival signal several articles presented that CD40 stimulation decreased the caspase-8 and caspase-3 activation, and the collapse of mitochondrial membrane potential but did not affect FADD-Fas association (Benson et al., 2006; Eeva et al., 2007).
2. The CD40-mediated upregulation of anti-apoptotic proteins delay the onset of the Fas-induced cell death. Both Flip short and long forms were upregulated in 4–24 h after CD40 stimulation and decrease thereafter reaching their lowest levels after 72 h (Hennino et al., 2000; Eeva et al., 2007). NFκB activation played crucial role on FLIP overexpression since its inhibition blocked the CD40-induced Flip overexpression (Hennino et al., 2000; Eeva et al., 2007). Results from different human cell lines showed that anti-apoptotic members of Bcl-2 family, Bfl-1/A1, and Bcl-xl, were upregulated by CD40. RNA of Bcl-xl and Bfl-1 are induced in 4–8 h following CD40 stimulation. The transcriptions of these anti-apoptotic proteins upon CD40 activation were completely abolished in the presence of constitutive IkB mutant, where exogenous Bcl-xl could restore the resistance to Fas-mediated cell death (Lee et al., 1999).

In the presence of CD40L different B cell lines and primary splenocytes upregulated Gadd45β expression in an hour, much earlier than the observable increase in the expressions of Bcl-xl and Flip. Overexpression of this protein, in the level comparable with normal cells upon CD40 activation (Zazzeroni et al., 2003), led to Fas resistance. Gadd45β inhibited the caspase activation and the mitochondrion depolarization, but neither the DISC formation, nor the early times Bid cleavage. These result suggest that Gadd45β inhibited the caspase activation following the activation of mitochondrial pathway (Zazzeroni et al., 2003).

### Adhesion, cell–cell contact

B cell contact either with T cells or with follicular dendritic cells influences Fas-induced cell death. In the case of antigen-specific T and B cell interaction, upon the formation of a T cell–B cell connection mediated by MHCII and the specific TCR, adhesion molecules reinforce T cell–B cell linkage, keeping the two cells in contact. Inhibition of the most prominent adhesion molecule pair, LFA1-ICAM1 by anti-LFA1 or an anti-ICAM1, completely blocked the cell death of target B cells (Wang and Lenardo, 1997) while the anti-CD2, anti-CD48 antibodies have weaker but significant effects. Because none of these antibodies directly affected anti-Fas-mediated lysis, the critical importance of the cell–cell contact time between the FasL-expressing T cells and Fas-positive target cells is suggested (Wang and Lenardo, 1997). Thus antigen-specific T cells fail to lyse B cells in the absence of long-term Fas-FasL signaling (Wang and Lenardo, 1997).

### B7.2

The expression of the prominent co-stimulatory molecule B7.2 is repressed in anergic B cells (Ho et al., 1994; Lenschow et al., 1994). In B7.2 transgenic mice, where the expression level of B7.2 in anergic B cells was restored and thus comparable with the level that is normally present on antigen-sensitive responding B cells, T cells did not kill the anergic B cells with appropriate antigen specificity (Rathmell et al., 1998). However, *in vitro*, B7.2 transgenic and normal anergic B cells were equally sensitive to anti-Fas-induced cell death. The survival effect of the co-stimulatory molecule was mediated by upregulated IL-4 production, which was only detectable upon the cooperation of antigen-specific T cells and B cells with high B7.2 expression and not in the case of B7.2 non-expressing B cells (Rathmell et al., 1998). In contrast, others found that blocking antibodies to B7.1 and B7.2 had no effect on CD4 T cell-induced cell death of normal, non-anergic B cells (Wang and Lenardo, 1997).

### FDC

GC B cells undergo apoptosis at least partially in a caspase-dependent manner when detached from their microenvironment (Lindhout et al., 1995). This process does not require any inducing death receptor signal, but Fas signaling accelerates this cell death route (van Eijk et al., 2001b). FasL-independent organization of preformed DISC and constant association of FADD, caspase-8, and FLIP_L_ with Fas were found in freshly isolated GC B cells. Accordingly, caspase-8 activation was observed within 40 min of the detachment of GC B cells without the need of Fas ligation (Hennino et al., 2001).

In the presence of FDC, GC B cells are protected from apoptosis, even after Fas activation both in mouse (Schwarz et al., 1999) and human systems (Lindhout et al., 1995; Koopman et al., 1997; van Eijk et al., 2001a), regardless of the fact that the presence of FDC enhances Fas expression in B cells (Tsunoda et al., 2000) and that FDC in the germinal center express FasL (Verbeke et al., 1999).

How the coculture with FDC is capable of inhibiting anti-Fas-generated cell death in B cells is not fully understood, but FLIP seems to be the crucial actor in this process (Schwarz et al., 1999).

### IL-4

Beside direct cell–cell contact, B cell survival is regulated by interleukins. Addition of IL-4 to CD40L-stimulated B cells reduces Fas-induced apoptosis (Foote et al., 1996a, 1998; Koizumi et al., 1996; Nakanishi et al., 1996; Wurster et al., 2002). The IL-4-generated resistance was synergistic with CD40L mediated survival (Nakanishi et al., 1996). Using suboptimal doses of anti-IgM, IL-4 was found to be strongly synergistic with BCR-transduced rescue signals (Foote et al., 1996a). However, the signaling mechanism utilized by IL-4 for Fas resistance differed from that used by IgM: first, the time required for IL-4-induced survival signal was longer than the one mediated by BCR (12–24 h); second, the IL-4 driven pathway whereas PKC independent (Foote et al., 1996a) absolutely required STAT6; third, through STAT6 activation IL-4 directly upregulated the Bcl-xl level (Wurster et al., 2002). One can note that some authors detected only marginal IL-4-induced upregulation of Bcl-xl (Schneider et al., 1997).

Unfortunately the CD40-independent survival potential of IL-4 signaling has not been yet investigated. From this study, the effect of overproduced Bcl-xl cannot be dissociated from the elevated FLIP level due to CD40 signaling. Upon upregulation of FLIP by BCR or CD40 signaling, Bcl-xl can clearly inhibit the Fas-mediated cell death pathway. However, the question remains as to whether the IL-4-induced upregulation of Bcl-xl alone is able to reduce Fas-induced cell death in B cells. Nonetheless, the most important consequence of this effect is that IL-4 transduced survival signaling, as non-antigen-specific signaling was not impaired in tolerant B cells while the BCR-induced survival signal did not function in these cells (Foote et al., 1998). The IL-4 signaling, by inhibiting the mitochondrial pathway, is able to keep the anergic B cells alive.

### CpG

Hypomethylated bacterial DNA contains a high percentage of CpG motifs that activate 95% of resting B cells. CpG containing oligonucleotides were found to effectively protect CD40L-stimulated B cells from CD4+ T cell-mediated apoptosis but had no effect on IgM-induced resistance (Wang and Lenardo, 1997). The CpG-triggered signaling pathways have not been extensively characterized (Table 2). Coligation of TLR9 and BCR promotes auto-antigen-specific B cell responses (Chaturvedi et al., 2008). This signaling pathway can break BCR tolerance and thereby rescue autoreactive but anergic B cells from Fas-mediated apoptosis.

## Further Questions

Our knowledge about the role of Fas in T-independent B cell responses is very limited. In the absence of T cell–B cell interaction, the potential for Fas-FasL interaction remains unknown. However the control of activated B cells is also crucial in this type of response. The B1 cell population has been suggested as FasL targets (see Table 1), but the source of FasL is still unknown.

As long-term T cell–B cell interaction requires common antigen-specificity, self-reactive B cells are probably not killed by T cells specific for external antigen. The role of Treg cells would be a very interesting aspect of MHCII-Fas interaction. Treg cells are key actors of peripheral tolerance to self-antigen. The expression and function of FasL was established in this immunosuppressive cell population (Baatar et al., 2007). Treg cells may eliminate autoreactive B cells through prolonged FasL-Fas triggering due to direct MHCII-TCR interaction (Janssens et al., 2003). Otherwise, in the absence of prolonged self-reactive B cell–T cell interactions, the killing of self-recognizing B cells, and the elevated number of autoimmune B cells in lpr mice would not be explained.

Fas aggregation, raft localization, and cytoskeleton association may be the targets of a survival pathway but little has been published about the BCR-mediated, protein-synthesis-independent, regulation of Fas signaling. Some authors have reported that BCR-mediated survival was absolutely dependent on protein synthesis (Foote et al., 1996b; Schneider et al., 1997) while others using the A20 cell line found that this effect was almost fully incomplete (Catlett and Bishop, 1999; Hinshaw et al., 2003). Based on Bras theory (Bras et al., 1997) two independent, but additive pathways, a prompt PKC-regulated and a time dependent NFκB directed, protein-synthesis-dependent pathway may collaborate in BCR-mediated survival. PKC could inhibit the very early, necessary step of Fas activation. Receptor aggregation (in Jurkat cells; Ruiz-Ruiz et al., 1999) and PKCζ was mentioned as a component of the DISC with inhibitory capacity (Leroy et al., 2005). A similar mechanism is supposed for B cells, explaining the reduced DISC formation upon BCR-induced PKC activation.

## Conflict of Interest Statement

The authors declare that the research was conducted in the absence of any commercial or financial relationships that could be construed as a potential conflict of interest.
